# Robust formation of optimal single spheroids towards cost‐effective *in vitro* three‐dimensional tumor models

**DOI:** 10.1002/2211-5463.13614

**Published:** 2023-05-05

**Authors:** Kinana Habra, Joshua R. D. Pearson, Stéphanie E. B. McArdle

**Affiliations:** ^1^ Chemistry department, School of Science and Technology Nottingham Trent University UK; ^2^ John van Geest Cancer Research Centre, School of Science and Technology Nottingham Trent University UK; ^3^ Centre for Health, Ageing and Understanding Disease, School of Science and Technology Nottingham Trent University UK

**Keywords:** 3D single spheroid, breast cancer, carnosine, glioblastoma, prostate cancer, U87 MG

## Abstract

While useful for fundamental *in vitro* studies, monolayer cell cultures are not physiologically relevant. Spheroids, a complex three‐dimensional (3D) structure, more closely resemble *in vivo* tumor growth. Spheroids allow the results obtained relating to proliferation, cell death, differentiation, metabolism, and various antitumor therapies to be more predictive of *in vivo* outcomes. In the protocol herein, a rapid and high‐throughput method is discussed for the generation of single spheroids using various cancer cell lines, including brain cancer cells (U87 MG, SEBTA‐027, SF188), prostate cancer cells (DU‐145, TRAMP‐C1), and breast cancer cells (BT‐549, Py230) in 96‐round bottom‐well plates. The proposed method is associated with significantly low costs per plate without requiring refining or transferring. Homogeneous compact spheroid morphology was evidenced as early as 1 day after following this protocol. Proliferating cells were traced in the rim, while dead cells were found to be located inside the core region of the spheroid using confocal microscopy and the Incucyte^®^ live imaging system. H&E staining of spheroid sections was utilized to investigate the tightness of the cell packaging. Through western blotting analyses, it was revealed that a stem cell‐like phenotype was adopted by these spheroids. This method was also used to obtain the EC50 of the anticancer dipeptide carnosine on U87 MG 3D culture. This affordable, easy‐to‐follow five‐step protocol allows for the robust generation of various uniform spheroids with 3D morphology characteristics.

## Graphical abstract

In the protocol herein, a rapid and high‐throughput method is discussed for the generation of single spheroids using various cancer cell lines in 96‐round bottom‐well plates. The method is associated with significantly low costs per plate without requiring refining or transferring. Homogeneous compact spheroid morphology was evidenced as early as 1 day after following this protocol.

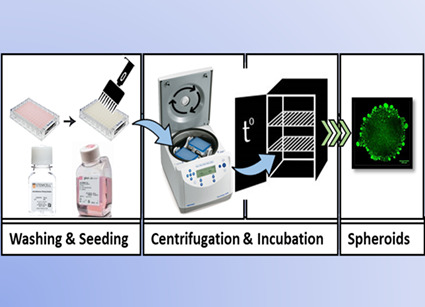

**Abbreviations**
3D: three‐dimensional
BCA: bicinchoninic acid
CFSE: carboxyfluorescein succinimidyl ester
DAPI 4′: 6‐diamidino‐2‐phenylindole
DMEM: Dulbecco's Modified Eagle Medium
EC50: half‐maximal effective concentration
EDTA: ethylenediaminetetraacetic acid
EMT: epithelial‐to‐mesenchymal transition
GBM: glioblastoma multiforme
H&E: hematoxylin and eosin
Opti‐MEM: reduced serum medium
PBS: phosphate buffer solution

Three‐dimensional models have been shown to have many advantages over monolayer cell systems. Spheroids have attracted attention due to their complex microenvironment and behavior, which mimic many natural *in vivo* conditions [1]. While single spheroids cannot replace scientific investigations of *in vivo* models, they represent a valuable bridge between monolayer cell studies and the more complicated structure of *in vivo* tumors [2]. The potential for high‐throughput investigations of anticancer treatments has been shown by spheroid formation [3]. The current standard methods for spheroid generation include the liquid‐overlay [4], hanging‐drop [5], and shaking methods [6, 7]. The limitations of these methods were found in the amount of spheroid formation and the relatively high costs. Some commercially available examples are Corning^®^, Costar^®^, and Brand^®^ ultra‐low attachment 96‐ or 24‐well plates and the Kuraray^®^ multiple pore types, all of which have the same basic principle. Other methods, such as magnetic levitation [8], NASA Bioreactor [9], and micro‐cages, require expensive tools. At the same time, the uniformity of the generated spheroids remains low [10]. Therefore, a method in which the reproducibility of forming copious quantities of uniform spheroids would be combined with the ability to keep costs low is required. Herein, we describe a detailed, cost‐effective protocol for establishing an *in vitro* 3D single spheroid model that can be used to identify potential new therapeutic approaches. We have shown that this method can be applied to many human and murine cancer cell lines of different origins (e.g., prostate cancer, TNBC, and GBM). More specifically, we have shown that the formation of spheroids (i.e., when the cells stop shrinking and start to grow) is cell‐type dependent and will need to be optimized for each cell line; however, spheroid formation was successful in all of the cell lines tested. These spheroids can then be utilized to investigate targeted drugs, antibodies, and immunoconjugates [11]. In addition, using U87 MG cells, a conventional glioblastoma cell line most studied due to its tumor stem cell‐like features [12, 13, 14] as an example, we generated single spheroids and assessed the effect of carnosine. This antitumor dipeptide has the potential to be used as a sustained release therapy for glioblastoma [6, 11, 15, 16]. Various chemotherapeutic agents have previously been studied using spheroids. In this paper, carnosine was chosen in this study due to its cancer‐specific toxicity [17].

## Materials and methods

### Cell culture

The human glioblastoma U87 MG‐Red‐FLuc cells (Bioware Brite, PerkinElmer, Beaconsfield, UK) were incubated in Opti‐MEM Reduced Serum Medium (Gibco™, Thermo Fisher Scientific, Loughborough, UK) culture medium, supplemented with fetal bovine serum up to 10%. The antibiotic puromycin (Gibco^®^, Thermo Fisher Scientific) was added after the initial thaw at 2 μg·mL^−1^. The incubation was at 37 °C in a humidified atmosphere containing 5% CO_2_. Other cell lines were cultured in their specific media by following the same protocol outlines. The human Glioblastoma SEBTA‐027 (Recurrent GBM cell line derived from the right parieto‐occipital region of a 59‐year‐old female) and SF188 (GBM cell line derived from an 8‐year‐old male) were cultured in Gibco™ DMEM, high glucose, GlutaMAX™ Supplement, and 10% fetal calf serum (Gibco™, Thermo Fisher Scientific). Both cell lines were a generous gift from the University of Portsmouth, neuro‐oncology group. The human Prostate cancer DU‐145, HTB‐81™ (American Type Culture Collection ATCC, Teddington, UK) were cultured in Eagle's minimum essential medium modified to contain Earle's balanced salt solution, nonessential amino acids, 2 mm L‐glutamine, 1 mm sodium pyruvate (BioWhittaker^®^ Medium EMEM Cell Culture Media, Lonza, Cologne GmbH, Germany), and 10% fetal calf serum. The murine prostate cancer TRAMP‐C1 (C57Bl/6 mice cells, which are derived from prostate adenocarcinoma cells from TRAMP mice) were cultured in Dulbecco's Modified Eagle Medium 4.5 g·L^−1^ glucose w/L‐Gln w/ sodium pyruvate (DMEM, Lonza, and 10% fetal calf serum. This cell line was provided by Matteo Bellone (University of Milan, Milan, Italy). The human breast cancer BT‐549 is ductal carcinoma (American Type Culture Collection ATCC) was cultured in Corning RPMI 1640 Medium with L‐Glutamine (Corning™ RPMI 1640 Medium, Corning, NY, USA), 10% fetal calf serum and 0.023 U·mL^−1^ insulin. The murine breast adenocarcinoma Py230 (American Type Culture Collection ATCC) was cultured in Corning Medium F‐12K with L‐glutamine (BioWhittaker^®^ Medium F12K Medium, Lonza), and 10% fetal calf serum, 0.1% MITO+ serum extender (Corning^®^).

### The protocol of spheroids generation

1. A volume of 50 μL of antiadherence rinsing solution (STEMCELL Technologies, Cambridge, UK) was added to each well of a 96‐well round bottom hydrophobic tissue culture plate with a growth surface for suspension (Green code: 83.3925.500, Sarstedt, Nümbrecht, Germany) [18].
2. After 15 min, the solution was discarded then each well was washed with 50 μL of serum‐free media.
3. The cells were grown as a monolayer and when the cells reached 70–80% confluence, they were sub‐cultured using 0.05% Trypsin−0.53 mm EDTA (Sigma‐Aldrich, St. Louis, MO, USA) for cell detachment.
4. To generate a single‐cell suspension, the cells were seeded (400 cells per well/100 μL full media).
5. Directly, the spheroid formation was initiated by centrifuging the plates with a benchtop centrifuge (Eppendorf 5810R Centrifuge, Hamburg, Germany) at 2710 ***g*** Force (rcf) for 10 min.

The plates were incubated between 1 and 12 days under standard cell culture conditions at 37 °C, and 5% CO_2_ in humidified incubators. The full media had been replaced each other day and postseeding cellular phenotype was checked by the microscopical observable physical properties of each spheroid including the appearance, development, and behavior.

### Localisation of dead and proliferating cells within spheroids

The seeded cells were stained by Incucyte^®^ Cytotox red for counting dead cells (250 nm, Essen Bioscience, Ann Arbor, MI, USA), and the generated spheroids were transferred to the Incucyte^®^ Live‐Cell Analysis System (Essen Bioscience Inc.). Live images were snapped with 4× objective lenses in each well every hour inside an incubator over 12 days. The culture medium was replaced every 2 days for maintaining the spheroid's survival. The localisation of the red dead cells within the spheroids was assessed by phase‐contrast images using the red channel to evaluate the real‐time cell membrane integrity and cell death. The total phase and the red fluorescent areas with mask were quantified for different days using incucyte ^®^ spheroid analysis software (Version. 2020B, Essen Bioscience Inc.).

### Imaging studies

The single spheroids were stained following the instructions of each kit. The dead cells were stained by Incucyte^®^ Cytotox red for counting dead cells (250 nm, Essen Bioscience) during the generation process. The cyanine nucleic acid dye permeated cells with compromised cell membranes. The green dye CFSE Cell Division Tracker Kit (BioLegend, London, UK) and DAPI (Sigma‐Aldrich) were used to stain the live cells and the nucleus before taking pictures. The morphology of the spheroids was assessed and recorded using the Incucyte^®^ Live‐Cell Analysis System and confocal laser scanning microscope (Leica, Wetzlar, Germany) by a 5× objective using the following settings: sequential scanning, ex/em: Mitotracker: 543/599 nm, Hoechst: 405/461 nm. The size of the spheroids was analyzed using Incucyte^®^ spheroid analysis software (Version. 2020B, Essen Bioscience Inc.), and image Software (Version. 1.44, National Institute of Mental Health, Bethesda, MA, USA).

### Hematoxylin and eosin H&E stain of spheroid cross‐sections

U87 MG spheroids were generated from 400 cells. Prior to staining, spheroids were cultivated individually after 1, 3, 5, 7, and 10 days washed once in phosphate‐buffered saline [Corning^®^, Phosphate Buffer Solution (PBS)] then fixed with 10% formalin (Merck, Darmstadt, Germany). The spheroids were washed in PBS and transferred to disposable biopsy embedding molds to encapsulate them with Epredia™ HistoGel™ Specimen Processing Gel (Thermo Fisher Scientific). Each gel‐coated spheroid was moved to a cassette and loaded inside a tissue processor (Excelsior AS, Thermo Fisher Scientific). The program was set to start with 6 times 70% ethanol, then 3 times xylene, and 3 times histology wax. Each cycle time was 10 min. Then, the capsules were embedded in paraffin using (Histostar, Thermo Fisher Scientific) with a temperature range of −3 to −12 °C. Microtone (Leica, RM2235) sections of 5 μm were placed on Super Frost glass slides (Menzel‐Glaser, Thermo Science, Germany) and allowed to dry for 2 h at 37 °C. The sections were deparaffinized by 2 changes of xylene for 5 min, rehydrated by 2 changes of 100% ethanol, followed by washing in 70% ethanol for 1 min. After a short single rinse in distilled water, the sections were stained for 20 min in Mayer's hematoxylin (Merck) and placed for 20 min under running tap water, then dipped in 1% Scott's Tap. The slides were observed under the microscope. Sections were counterstained with eosin (Merck) for 2 min, rinsed quickly in tap water, and dehydrated by a dip in 70% ethanol, followed by two changes in 100% ethanol for 2 min each and two changes of xylene for 2 min. The slides were mounted in DPX (Sigma‐Aldrich, Darmstadt, Germany), and left to air dry for 2 h. spheroid sections were assessed by brightfield microscopy.

### Immunoblotting

A confluent flask (70–80%) of U87 MG cells grown in monolayer culture served as a control. After 10 days, spheroids were pooled from the plate. All spheroids were washed twice with ice‐cold PBS (Phosphate Buffer Solution), then lysed in 500 μL RIPA lysis buffer (50 mm Tris–HCl pH 8, 150 nm sodium chloride, 0.1% sodium dodecyl sulfate (SDS), 0.5% sodium deoxycholate, 1% Triton ×100, 1 mm EDTA) containing protease inhibitor cocktail (Sigma, Gillingham, UK). The lysates were vigorously vortexed and placed on the ice every 10 min for a 30‐min period. After using the vortex mixer, the samples were centrifuged for 15 min at 25 000 ***g*** at a temperature of 4 °C. The protein concentration was determined by a BCA protein assay (Sigma). A total of 20 μg protein extract was mixed with 5× Laemmli loading buffer (50% glycerol, 10% SDS, 0.25% bromophenol blue, 250 mm Tris–HCl pH 6.8, 5% β‐mercaptoethanol) resolved on a 10% SDS/PAGE gel and a wet transfer was performed with 25 mm Tris, 192 mm glycine and 20% methanol for 90 min at 100 V onto a nitrocellulose membrane. Membranes were then blocked with a 5% milk in TBST solution and then probed with primary antibodies directed against β‐actin (Sigma), vinculin (Abcam, Cambridge, UK), vimentin, ZO‐1, or E‐cadherin (Cell Signaling Technologies, Leiden, Netherland). After incubation with primary antibodies, HRP‐linked secondary antibodies (Cell Signaling Technologies) were used to detect bound primary antibodies in combination with Clarity Western ECL substrate (BioRad Laboratories, Watford, UK). The intensity of the western blot bands was quantified using ImageJ software, and the expression of the protein of interest was expressed as the intensity of the detected band/the intensity of the beta‐actin or vinculin loading control band.

## Results

**Fig. 1:**
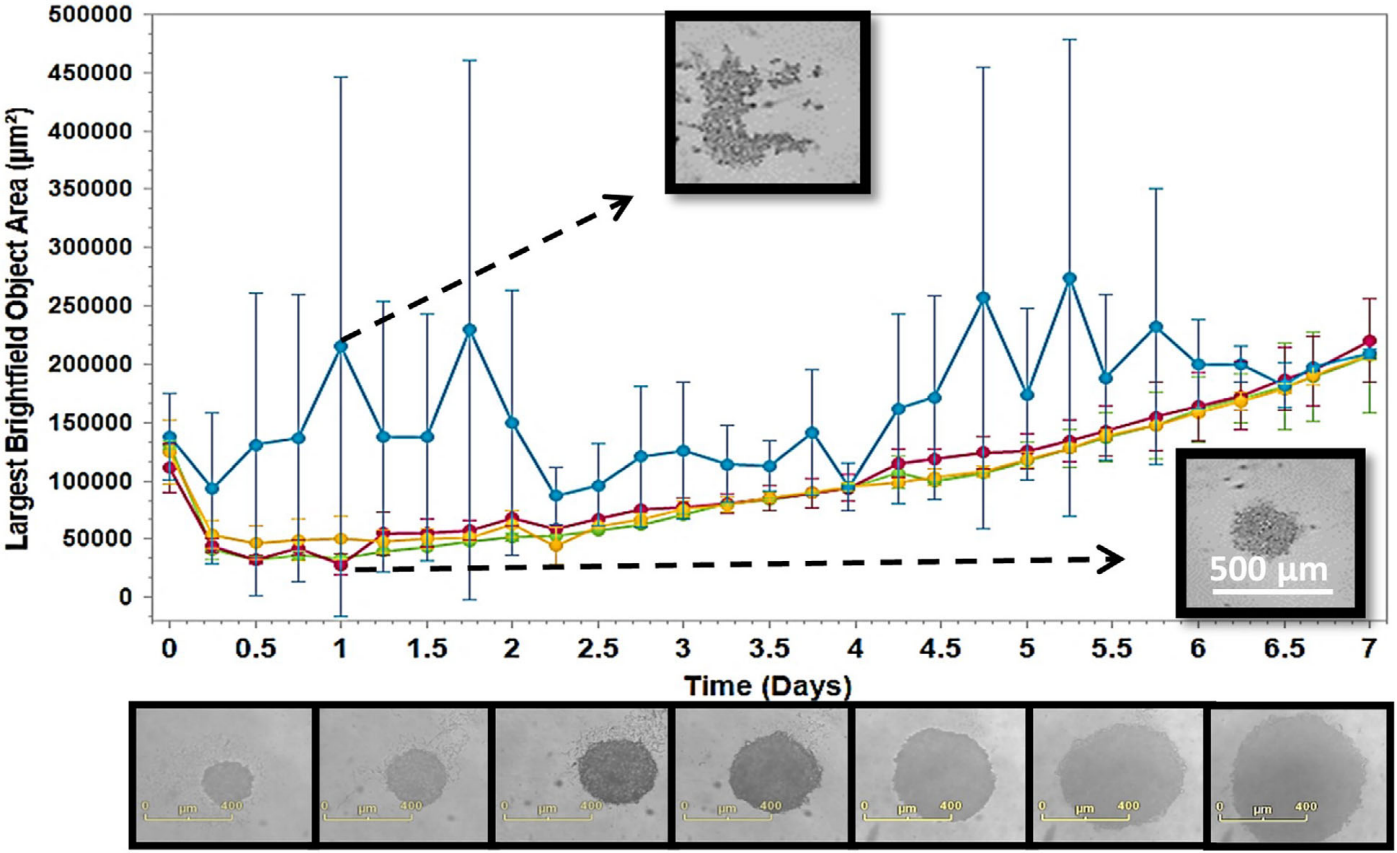
Incucyte^®^ Live‐Cell System (×4) live spheroids images analysis shows the proliferation curves of the confluence ratio of the U87 single spheroid upon using the protocol steps. The blue graph of the control showed the inability of the cells to convert the aggregates of cells into a spheroid without using the washing solution. Whereas consistent single spheroid formation reproducibility was achieved for the three different wells when the protocol was followed. The experiment was repeated on triplicate and the images show the difference between the shape of the aggregates and the successful shape of the spheroid, (scale bar = 500 μm). The series of images below the graph show the growth of the spheroids from days 1 to 7, (scale bar = 400 μm).

The protocol was applied to evaluate the homogeneity of the generated spheroids. The size distribution and mean confluence of the phase area for individual spheroids were quantified using incucyte ^®^ software [19]. The cells were seeded in three wells of a 96‐well plate. An additional well was used as a control (i.e., no washing). These were left to grow for 7 days to form spheroids while being monitored hourly. The results showed that following this protocol, the consistent spheroid formation was achieved with high reproducibility independently of the seeding well within the plate. The cells gathered over the first day, as detected by a reduction in the number of largest brightfield areas (Fig. 1). By contrast, the control cells failed to convert into a spheroid. Instead, aggregates were formed, highlighting the importance of the washing step as indicated in the blue graph in Fig. 1. After that, unlike the aggregates seen in the control well, a regular morphology with a uniform spherical geometry and a narrow size deviation was displayed by the spheroids. For U87MG cells, seeding 400 cells per well on day 0 was sufficient to convert them into homogeneous spheroids that ranged in diameter from 216 ± 9 μm after 1 day to 475 ± 8 μm after 5 days, and 847 ± 11 μm after 7 days. The homogeneity of the spheroids was reflected by a slight standard deviation, ranging from the mean in Fig. 1 and Fig. S1a(A).

In addition to washing, another critical factor to consider was the type of plate used. Indeed, using a 96‐well round bottom plate with a hydrophobic surface designed for suspension cells (Green code: 83.3925.500, Sarstedt) proved to be a critical step in promoting the formation of homogenous spheroids. By contrast, single spheroids could not be generated using a 96‐well round bottom with a standard growth surface for adherent cells (Red code: 83.3925, Sarstedt) despite washing the surface of the wells with the antiadherence solution. The cells adhered to the bottom of the wells, forming an incomplete circle after centrifugation, Fig. S1a(B,C). The most critical steps in the protocol were the use of the green code plate and the antiadherence washing solution, which consisted of an amphipathic component to prevent cell adhesion [20]. It was shown with this method that cells formed spheroids in every seeded well at various locations horizontally and vertically on the plate. The consistency and reproducibility of producing spheroids in the 96‐wells were confirmed by this monitoring (Fig. S1b).

The obtained spheroids were further characterized using the Incucyte^®^ Live‐Cell Imaging System. The 3D structure of the spheroids was shown to be achieved uniformly in all seeded wells. The spheroids grew consistently for up to 12 days. The overall transformation from forming aggregates to generating tight single spheroids where the dead cells (stained in red) were localized in the center, and the live cells proliferated at the rim of the spheroid. This was confirmed by the observed morphology. The live CFSE‐stained cells surrounding the Cytotox red color, which was taken up by dead cells at the core of the spheroid, were shown by the Incucyte images with a green channel filter. This has the advantage of assessing the mobility of the tumor cells, enabling the monitoring of the invasion of the U87 MG cells from the surface of the spheroid. The invasion area was estimated after applying an invasion mask and subtracting the area of the dead cells from the whole spheroid in Fig. S2a(E). A significant increase in growth from the fifth day, confirmed by the viability of the cells at the rim of the spheroid (CFSE green live cells and DAPI blue nucleus), was shown by confocal microscopic images of the spheroid morphology. The dark shade where the dead cells were located was demonstrated by the 5‐ and 10‐day spheroid images across the center (Fig. S2b).

**Fig. 2:**
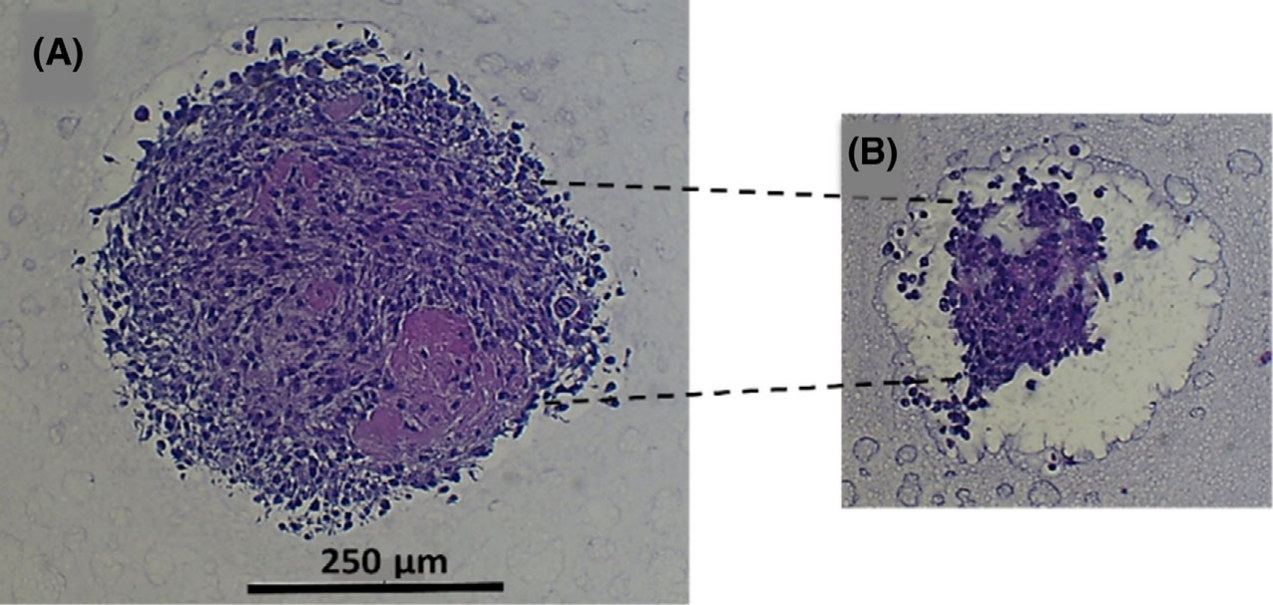
Cell tightness and interaction analysis of U87 MG spheroids show H&E stain of spheroid cross‐sections from (A) the core area and (B) the top rim area of the seven‐day spheroid generated from 400 cells, (scale bar = 250 μm).

Histological sections were examined for spheroids grown for 7 days in culture to investigate the tightness of the cell packaging in a single spheroid. H&E staining was applied to these spheroids after fixation and embedding. Hematoxylin stains cell nuclei with a purplish‐blue color, while eosin stains the extracellular matrix and cytoplasm pink. The H&E stain from the center and the rim cross‐section of a spheroid are shown in Fig. 2. With this, the cell density was high in the core region, whereas the daughter cells gathered around the rim to tighten and increase the spheroid size.

**Fig. 3:**
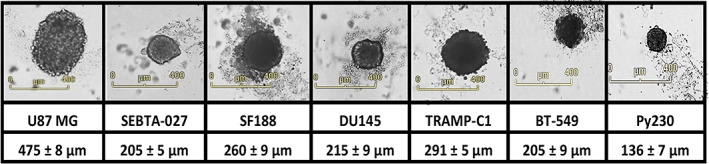
Incucyte^®^ Live‐Cell System (×4) live spheroids images show the potential of using the protocol to obtain single spheroids from various cell lines including brain cancer cells (U87 MG, SEBTA‐027, SF188), prostate cancer cells (DU‐145, TRAMP‐C1), and breast cancer cells (BT‐549, Py230), (scale bar = 400 μm). The measurement was obtained for the diameter on day 5.

Glioma is composed of a heterogenous type of cells, with some adopting a highly invasive mesenchymal phenotype, as opposed to an epithelial phenotype [21]. This epithelial‐to‐mesenchymal transition (EMT) was also previously identified in spheroids formed using CAL33 head and neck squamous cell carcinoma [22]. Similarly, we have found that 15 days postseeding, the U87 MG cells significantly increased vimentin and decreased both ZO‐1 and E‐cadherin (Fig. S3). These EMT changes are indicative of a cancer stem cell phenotype, a population known to be highly treatment‐resistant and responsible for tumor recurrence [23]. As a result of these findings, the proposed technique represents an excellent *in vitro* single spheroid model for testing potential therapies as a preliminary platform before moving to *in vivo* mouse models. The applicability and reproducibility of this method were demonstrated using different cell lines from various cancer types (originating from either humans or mice) (Fig. 3). All different cell lines showed similar morphology during the growth of single spheroids as shown previously in Fig. 1. The Video S1 is provided to represent an example for the live growth and daily measurements of the U87 MG spheroids over 7 days.

**Fig. 4:**
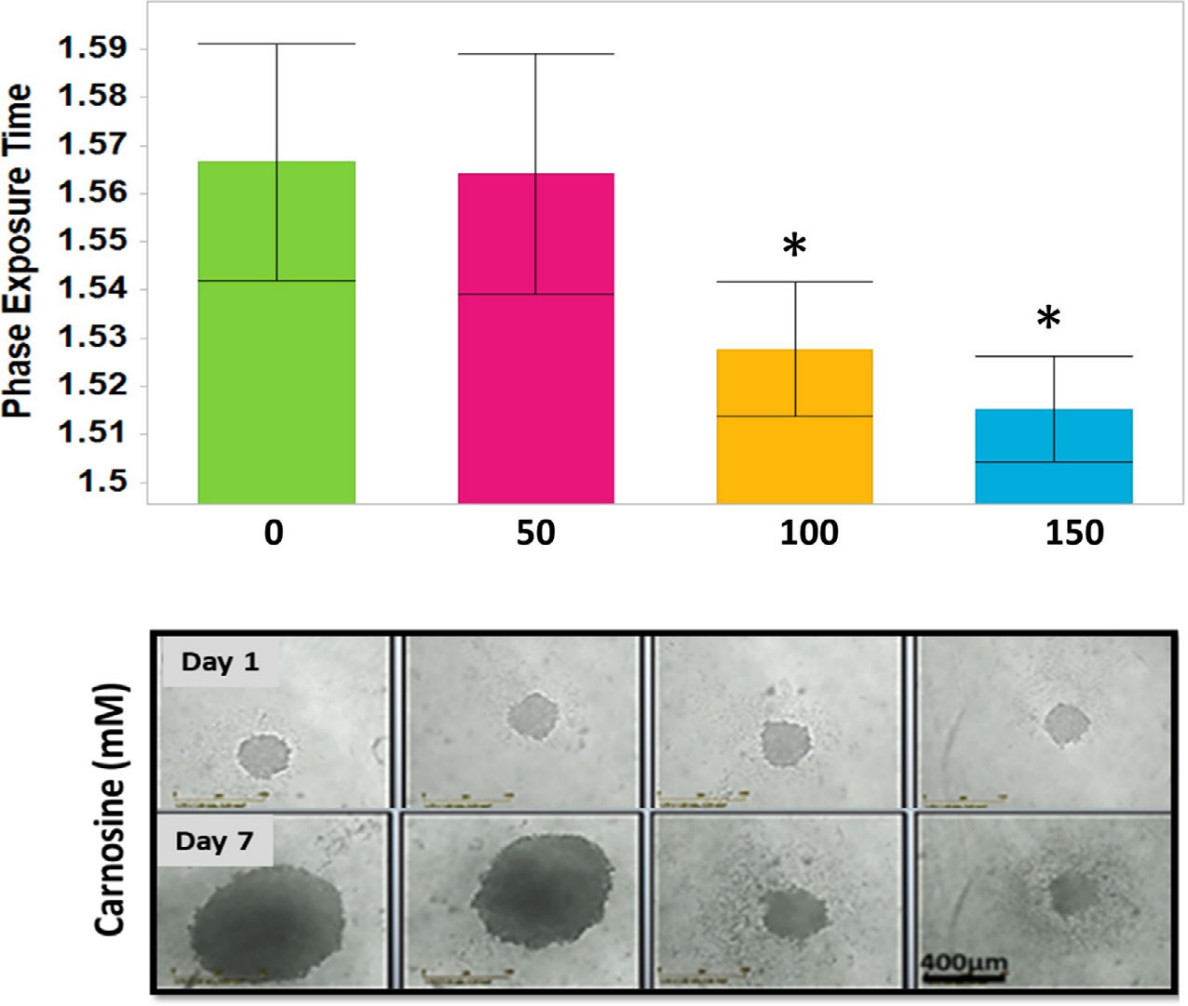
Incucyte^®^ Live‐Cell System (×4) live spheroids images show the comparison between the morphology and size of single spheroids on day one and day seven after applying frequent doses of carnosine treatment. The mimicked sustained release of carnosine concentration ≥ 100 mm affected spheroid growth and tightness, and a significant difference was reported in phase exposure. The one‐way analysis of variance (ANOVA) was carried out for multiple comparisons between the control and each group using the Dunnett's post‐test. The statistical significance level on day seven was indicated with (*) for *P* < 0.01. Error bars represent the standard error of the mean (SEM), *n* = 3, (scale bar = 400 μm).

Additionally, the efficacy of an antitumor dipeptide known as carnosine was measured by applying carnosine at different concentrations (e.g., 0, 50, 100, 150 mm) to U87 MG grown as single spheroids on days one, three, and five to mimic sustained release therapy. The change in morphology of the single spheroids after day three proved the effect of carnosine in suppressing the proliferation of these spheroids. From the series of carnosine concentrations, the critical amount to hinder spheroid growth was over 100 mm (Fig. 4).

**Fig. 5:**
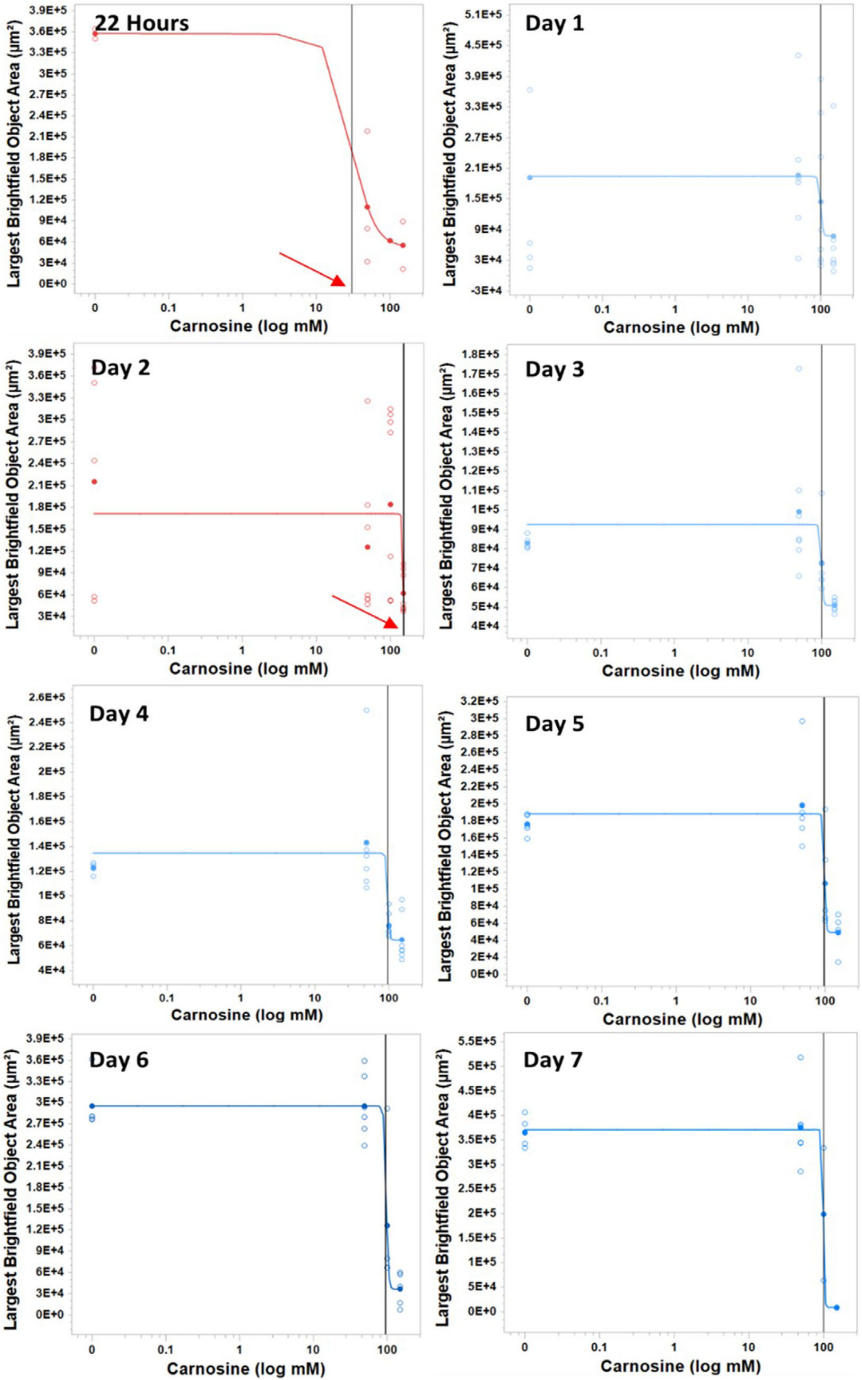
Incucyte^®^ Live‐Cell System (×4) live spheroids images show the potential of using the protocol to obtain EC50 of carnosine on multiple points for drug delivery study and *in vitro* sustained release profile. The black vertical line represents the EC50 of carnosine on spheroids. It was calculated on daily basis starting from the time of recognizing the spheroid until day seven. The arrows indicate the continuous increase in EC50 before adding the second dose. The blue graphs indicate EC50 = 100 mm. Last‐day readings showed zero size with the treatment of 150 mm of carnosine after mimicking the sustained release of carnosine treatment over a week.

In a previous investigation that utilized a monolayer 2D model, the concentration of carnosine required to affect the viability of U87 MG was found to be around 30 mm after 24 h [15]. We also found that the EC50 of carnosine was around 30 mm at 22 h (prior to cell aggregates forming tight spheroids) while the cells started to show the tightness of the spheroid on different speeds between day 1 and day 2 (Figs 1 and 5). The individual size of the control spheroids was similar after 22 h and from day 3 upward after the shrinking phase. However, the daily comparison of the drug sensitivity in 3D cultures was feasible by measuring EC50 using the change of spheroid largest brightfield object area. The spheroids, which were exposed to carnosine were not able to tighten like the control untreated spheroids. The increment of EC50 from 30 to 155 mm on day 2 was a critical indicator of the decrease in the carnosine effect. Thus, a carnosine dose from each concentration was added every other day. The EC50 showed stability around 100 mm until day seven, confirming the suitability of the dose frequency (Fig. 5).

## Discussion

Historically, theranostic anticancer compounds were most often applied to cells grown in monolayers. Over the past decade, sophisticated systems, such as 3D cultures, have been developed to improve the prediction of the clinical efficacy of compounds [24]. For example, Nunes et al. [25] formed 3D models of glioblastoma multiforme (GBM) tumors with tumor‐associated astrocytes, microglia, endothelial cells, and immune cells. Cell–cell interactions were reproduced by 3D models, simulating the tumor microenvironment. These complex cell–cell interactions are not present in 2D monolayer cell cultures, influencing drug response [25]. Many advantages and disadvantages of tumor spheroid formation have been addressed by popular methods [6, 26]. A similar vigorous intensity to our method was shown with ultra‐low attachment well plates, which have been used to form several types of human and murine tumor spheroids of various cell lines [6, 7, 27]. 3D tumor spheroid applications included U87 MG, SEBTA‐027, SF188, DU‐145, TRAMP‐C1, BT‐549, and Py230 cell lines.

We found it essential to use hydrophobic (non‐wettable) polymer 96‐well plates, as these plates made it difficult for cells to attach to the bottom of the well. The red‐coded plates are modified to have a hydrophilic surface, allowing cells in serum‐containing culture medium to adhere and spread on the bottom of the well. However, the green‐coded plates with unmodified polystyrene minimize the surface attachment effect and cell adherence [28, 29]. Furthermore, coating the surface of the wells with a film of zwitterionic material effectively reduces or eliminates nonspecific adsorption of the cells to the solid interface [20]. Cells were allowed to assume a much rounder cylindrical morphology during the centrifuging process compared with the flatter morphology typically observed in the vessels. Seeding at a density of 400 cells per well using 100 μL full media was optimized to obtain a spheroid size of around 200 nm. Spheroids with a large average diameter of > 200 μm are exposed to a large shear force. As a result, it is impossible to form stable spheroid‐spheroid connections [6].

Moreover, we found that the speed of centrifugation played an essential part in forming tight spheroids, as too low a centrifugation speed led to the formation of aggregates [30, 31]. This allowed the cell–cell connection to be retained while inhibiting the cell‐well connection by the zwitterionic liquid wash. Since the plates were under orbital rotation, cell collection was induced in the center of each well with even distribution by centrifugal forces [32]. Cell maturation was monitored using the Incucyte^®^ live imaging system, which allowed viewing of the regular changes in each well. Incucyte^®^ spheroid software or z‐stacks may be used to image large single spheroids and avoid false‐negative data [33]. In a previous report, the liquid‐overlay method was performed on a 96‐well plate, and the equivalent diameter (range, mean ± SD, CV%, *n*) of the obtained spheroids on day seven was found to be within the range of 275–350 μm (mean 312 ± 23 μm, CV of 7.37%, *n* = 32) [27]. These results were close to those obtained with the shaking separated cell sheet method within dispase‐doped media after 9–12 days, seeding 800 cells per petri dish at the start of the experiment. Indeed, the spheroids were found to have sizes ranging from 172 to 241 μm (mean 201 ± 13 μm, CV of 6.35%, *n* = 460) [6]. Despite the high number of harvested spheroids from other methods, the protocol detailed herein is better regarding the amount, uniformity, lowest deviation, and fastest preparation. Within 2 days and starting with 400 cells/100 μL per well using a 96‐well plate, the diameter of the harvested spheroids had a size range of 155–259 μm (mean 216 ± 9 μm, CV of 4.16%, *n* = 63).

An easy method for mass production of homogeneous and uniform 3D cultures would lead to a highly efficient sorting process that minimizes both setup time and wasted 3D cultures [34]. Utilizing the Incucyte^®^ system and confocal imaging for 3D image analysis enabled the characterization of the 3D cellular matrix of different spheroid phenotypes. The spheroids were characterized by studying the 3D structures, cell viability, and necrosis. The presence of dead cells in the spheroid center was due to hypoxia [35]. Tumor hypoxia has been attributed to tumorigenesis and therapeutic resistance by maintaining the undifferentiated state of tumor stem cells. Therefore, therapeutic strategies should take oxygen tension into account [35].

The loss of green CFSE fluorescent signal throughout the z depth of the spheroids exhibited a reproducible exponential decay function [36]. Monitoring the changes in the content of live, dead, and apoptotic cells enables the observation of the consequences of compound exposure on the spheroid [37]. The H&E staining of the middle cross‐section of the spheroid encompassed complete tight structures from core to rim. The rim of the spheroid consisted of even layers of packed cells toward the center, despite the death of the cells, which formed a necrotic core region [38]. During spheroid formation, a small proportion of cells did not integrate into the sphere and lose cell–cell adhesion properties. The reason for this separation is gravity‐sedimentation [39]. It was revealed by western blotting that spheroids generated after 10 days of culture downregulated their expression of vimentin, albeit not significant when compared to cells grown in monolayer. The downregulation of vimentin further indicates that the cells within these spheroids appear to adopt a stem cell phenotype. The procedure employed was confirmed by the previous validation process to be suitable for its intended use. The reported results addressed the quality, reliability, and consistency of optimal *in vitro* 3D model generation relying on a robust and cost‐effective protocol.

The penetration and binding of compounds into spheroids are promising predictors of compound uptake in thick tissues [40]. The formed tumor spheroids of U87 MG reflected the effect concentration EC50 by using carnosine as a treatment [41]. By utilizing different concentrations (e.g., 0, 50, 100, and 150 mm) of carnosine in a sustained release designed experiment, the inhibition of the growth of single spheroids was significant compared with untreated spheroids [15]. The potential of this method for generating spheroids from various cancer types (e.g., brain, prostate, and breast), starting with the same cell density per well, was shown with the generation of single spheroids from seven different murine and human tumor cell lines. The size of the spheroids can be adjusted by seeding different cell numbers and manipulating the incubation time according to the personalized experiment design.

However, one should remember that the core region, consisting of a necrotic area, will also increase with the spheroid size. Therefore, experiments aiming to assess treatment efficacy must be carefully planned [42]. After optimizing toward mimicking various stages of avascular tumor regions, the resulting single spheroids can easily be transferred to any plate or cell culture vessel due to the ease of mechanical access for further investigation or analysis. Cell viability assays, such as MTT, trypan blue exclusion, and LDH release assays, can be used for *in vitro* therapeutic screening in spheroids [24]. Consistent culture conditions need to be maintained during spheroid growth. Otherwise, proliferation may be significantly affected by this, altering the expression of tight junction molecules and establishing a delay in the initial shrinking of the spheroid size. On average, a stable symmetrical size is reached by the spheroids 24–48 h postseeding. However, some cell lines require a longer time, such as the DU‐145 cells, which require around 5 days to produce firm spheroids. The observed diameter was *ca*. 200 nm, at which point the spheroids started to show a necrotic core and a proliferative outer layer. In future, adding cells of the tumor microenvironment to develop multicellular 3D cultures will make the models more representative of the *in vivo* tumor situation [43]. This protocol could also be tested on essential tumor stem cells such as SJ‐1 [35]. Importantly, spheroids will form using various cell densities and we recommend that the researcher test different ones in order to choose the one that would most suit the need of their experiment. This density used in this paper was so that all the cell lines studies would form spheroid between 3 and 7 days. Clonal evolution is a phenomenon known to occur in tumor cells; nonetheless, we have been able to use this method to form single spheroid using both high and low passage numbers; however, we have not genetically characterized them and therefore cannot confirm whether clonal evolution did not occur. If clonal evolution has occurred it does not seem to affect the ability of the cells to form single spheroid.

## Conclusion

Monolayer‐cultured tumor cells have less resistance to therapeutic interventions than *in vivo* cells. Developing a 3D model resembling solid tumors is essential, especially when bridging the gap between *in vitro* and *in vivo* tumor models. The use of 3D models is now largely diffused, and significant updates follow the commonly used protocols. The protocol described here demonstrated reproducible findings in generating robust single spheroids using a simple, cost‐effective method from which other researchers and laboratories can benefit. Single uniform spheroids without any additives were consistently obtained. The spheroids exhibited a typical characteristic morphology consisting of a proliferating rim and a necrotic core. These can then be used to assess the EC50 of drugs, as was highlighted using carnosine treatment. Preliminary optimization for different cell lines of single spheroids is proposed to provide the research workers with an easily accessible and average 20‐fold cheaper method than the ultra‐low adherent plates for *in vitro* investigation. In future, the reported data need to be further studied to find the validity of patient‐derived tumor cells.

## Conflict of interest

The authors declare no conflict of interest.

## Author contributions

KH contributed to experimental design, performed the experiments, and wrote the first draft, JRDP performed the western blot experiment and contributed to the final manuscript, and SEBM contributed to the final manuscript. All authors have read and agreed to the published version of the manuscript.

## Supporting information


**Fig. S1.** Incucyte^®^ Live‐Cell System (×4) live spheroids images analysis. (a) A. The proliferation curves of individual U87 single spheroids from individual locations in the 96‐well plate (C2, C3, D2, D3). The spheroid area decreased during the first day. Then, each tight spheroid grew over seven days. B. The instant adherence of the cells when skipping the step without washing. C. Represent the cells in different wells as referred in well A. The cells were gathered after washing with the anti‐adherence solution with a 96‐well round‐bottom standard growth surface for adherent cells (red code). However, a crack appeared in the gathered cells after centrifugation. In the B and C cases, the cells aggregates. (b) The U87 cells in the 96‐well plate image turned into spheroids in various horizontal and vertical in the center of each seeded well after one day, with a size distribution of 216 ± 9 nm for individual spheroids. The figure shows that spheroids were not randomly growing in different locations which reflects the reproducibility in all wells.

**Fig. S2.** Fluorescence cell Imaging. (a) Incucyte^®^ live‐cell system (4×). Live spheroid images after 10 days. A. The confluence of the live cells around the spheroid is shown by the phase image to be distributed uniformly. B. The localization of the dead cells in the center of the spheroid is shown by the phase with a red channel filter image. C. The 3D localization of the live cells around the spheroid is shown by the phase with a green channel filter image. D. The 3D shape of the dead/live cells in the same spheroid is shown by the overlap of the red and green channels. E. The mask of the invasion area. (b) The distribution of the live green cells (CFSE) and the blue nucleus (OAPI), which are located close to the rim of the spheroid, are shown by confocal images. A & C. The dark shade of the dead cells is shown by the 5‐ and 10‐day spheroid images across the center S & D, The 5‐ and 10‐day spheroid images of the live cells from side projection across the rim area.

**Fig. S3.** Western blotting analysis of EMT markers expression. (a, c and e) Western blot results for (a) E‐cadherin and beta‐actin, (c) ZO‐1 and beta‐actin and (e) Vimentin and Vinculin obtained from U87MG cells grown either in a monolayer or as spheroids generated from 400 cells for 15 days. (b, d, and f) Quantification of the protein tested relative to either beta‐actin or vinculin as detected by western blotting of lysates from monolayer and spheroid cultures across three different passages. Error bars = SD, n= 3, * = p < 0.05, ** = p < 0.01.

**Video S1.** Incucyte^®^ Live‐Cell System (×4) live spheroids video analysis shows live spheroid growth after seeding.

## Data Availability

The data presented in this study are available on request from the corresponding author.
