# Supplementary figures and images for: Robust formation of optimal single spheroids towards cost‐effective in vitro three‐dimensional tumor models
Source: FEBS Open Bio. 2023 May 5;13(7):1266–77. doi: 10.1002/2211-5463.13614 (PMC10315810; doi:10.1002/2211-5463.13614)

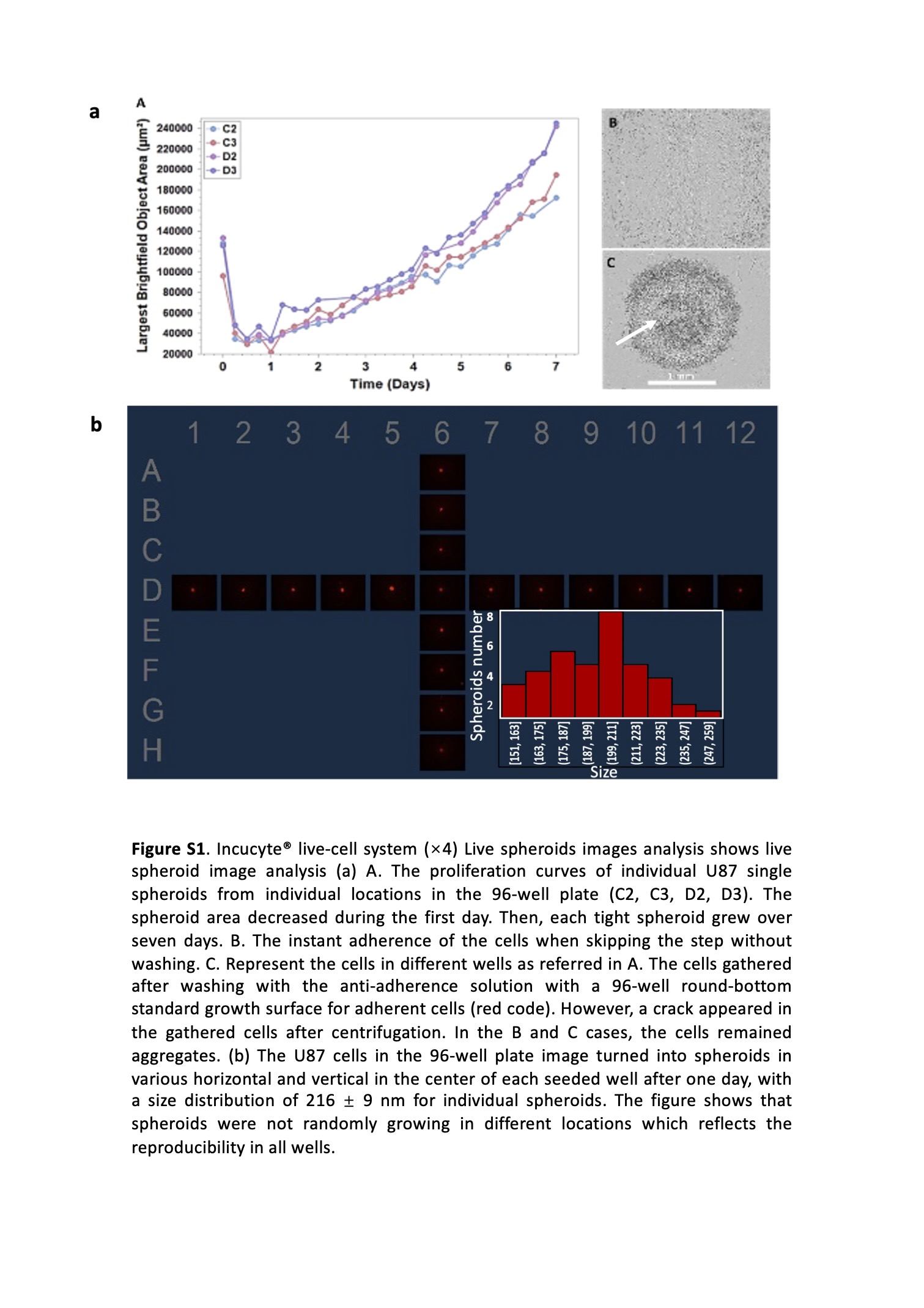

Supplement: Supplementary file 1 — Fig. S1. Incucyte® Live‐Cell System (×4) live spheroids images analysis. (a) A. The proliferation curves of individual U87 single spheroids from individual locations in the 96‐well plate (C2, C3, D2, D3). The spheroid area decreased during the first day. Then, each tight spheroid grew over seven days. B. The instant adherence of the cells when skipping the step without washing. C. Represent the cells in different wells as referred in well A. The cells were gathered after washing with the anti‐adherence solution with a 96‐well round‐bottom standard growth surface for adherent cells (red code). However, a crack appeared in the gathered cells after centrifugation. In the B and C cases, the cells aggregates. (b) The U87 cells in the 96‐well plate image turned into spheroids in various horizontal and vertical in the center of each seeded well after one day, with a size distribution of 216 ± 9 nm for individual spheroids. The figure shows that spheroids were not randomly growing in different locations which reflects the reproducibility in all wells. [file FEB4-13-1266-s001.jpg]

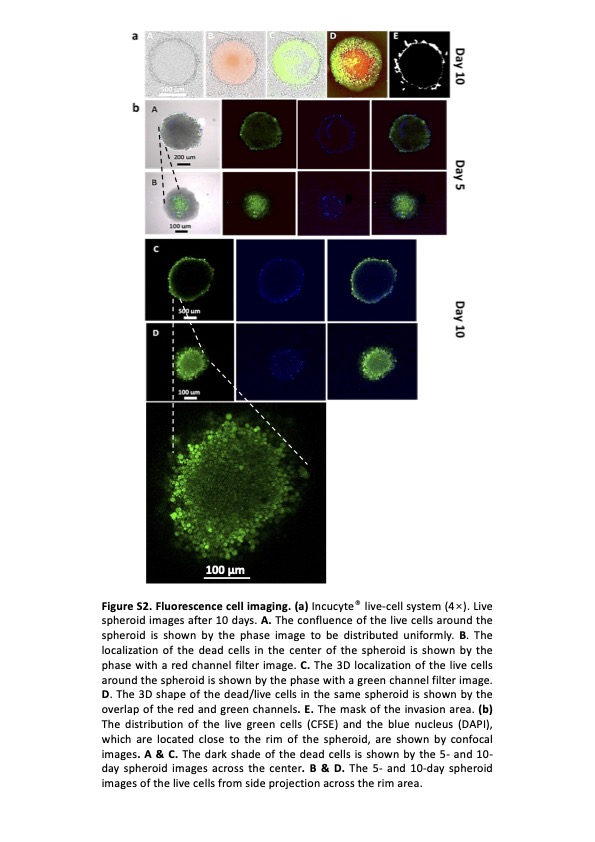

Supplement: Supplementary file 2 — Fig. S2. Fluorescence cell Imaging. (a) Incucyte® live‐cell system (4×). Live spheroid images after 10 days. A. The confluence of the live cells around the spheroid is shown by the phase image to be distributed uniformly. B. The localization of the dead cells in the center of the spheroid is shown by the phase with a red channel filter image. C. The 3D localization of the live cells around the spheroid is shown by the phase with a green channel filter image. D. The 3D shape of the dead/live cells in the same spheroid is shown by the overlap of the red and green channels. E. The mask of the invasion area. (b) The distribution of the live green cells (CFSE) and the blue nucleus (OAPI), which are located close to the rim of the spheroid, are shown by confocal images. A & C. The dark shade of the dead cells is shown by the 5‐ and 10‐day spheroid images across the center S & D, The 5‐ and 10‐day spheroid images of the live cells from side projection across the rim area. [file FEB4-13-1266-s004.jpg]

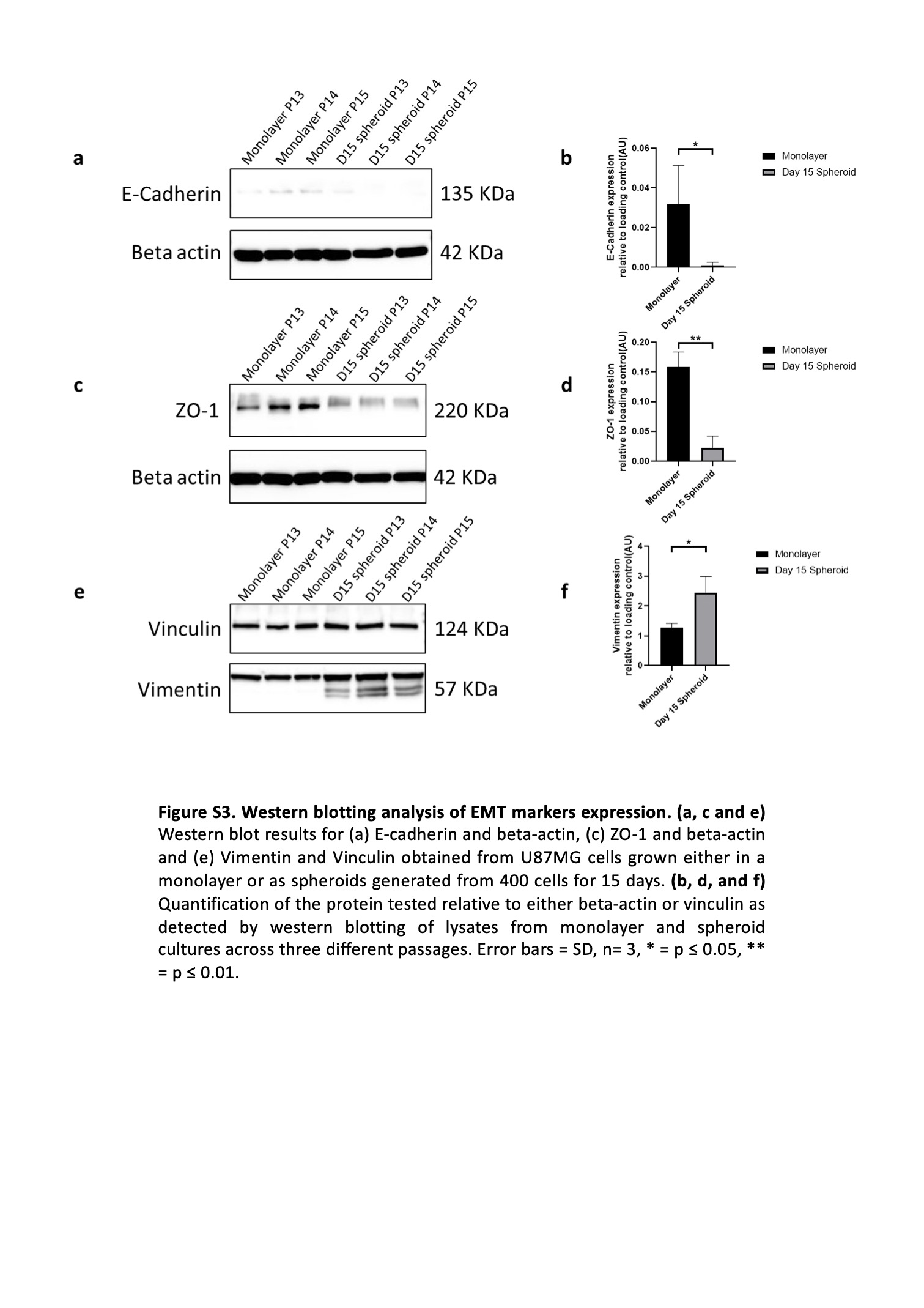

Supplement: Supplementary file 3 — Fig. S3. Western blotting analysis of EMT markers expression. (a, c and e) Western blot results for (a) E‐cadherin and beta‐actin, (c) ZO‐1 and beta‐actin and (e) Vimentin and Vinculin obtained from U87MG cells grown either in a monolayer or as spheroids generated from 400 cells for 15 days. (b, d, and f) Quantification of the protein tested relative to either beta‐actin or vinculin as detected by western blotting of lysates from monolayer and spheroid cultures across three different passages. Error bars = SD, n= 3, * = p < 0.05, ** = p < 0.01. [file FEB4-13-1266-s003.jpg]
